# Open surgical repair of an abdominal aortic aneurysm during the second trimester of pregnancy

**DOI:** 10.1016/j.jvscit.2024.101647

**Published:** 2024-10-22

**Authors:** Ndeye Fatou Sow, Abdoul Ahad Mbengue, Jean Michel Davaine, Morgane Lemierre, Papa Adama Dieng

**Affiliations:** a Thoracic and Cardio-Vascular Surgery Department, FANN University Hospital Center, Dakar, Senegal; b Vascular Surgery Department, CHU Pitié- Salpêtrière University Hospital, Paris, France; c Medicine Department, Sorbonne University, Paris, France

**Keywords:** Abdominal aortic aneurysm, Pregnancy, Open surgery, West Africa

## Abstract

A 37-year-old obese woman, trigravida and secundiparous, at 17 weeks of gestation presented with acute abdominal pain. Doppler ultrasound examination showed an infrarenal abdominal aortic aneurysm measuring 61 mm in diameter and a computed tomography angiography outlined a fissured infrarenal aneurysm measuring 85 mm in diameter. To preserve the pregnancy, the obstetrician performed perioperative tocolysis. Subsequently, open surgery was conducted using open repair with 60 minutes of infrarenal cross-clamping. The postoperative course was uneventful, and the patient was discharged on day 10. She subsequently gave birth to a healthy baby at full term.

Abdominal aortic aneurysm (AAA) during pregnancy is rarely described. It is a high-risk situation of maternal-fetal mortality. Pregnancy, by increasing the aortic diameter, exacerbates the risk of rupture of AAA.^1^ The reported etiologies primarily include connective tissue abnormalities (Marfan syndrome and related disorders) and infections. In rare cases, the etiology can be congenital, inflammatory, or remain undetermined.^2^^,^^3^ Therapeutic options, whether open surgery or an endovascular approach, may be complex and require a well-equipped facility and a skilled team.^4^^,^^5^ We present a case of a 37-year-old pregnant woman with a pre-ruptured AAA who was treated successfully with open surgery.

## Case report

**Fig 1:**
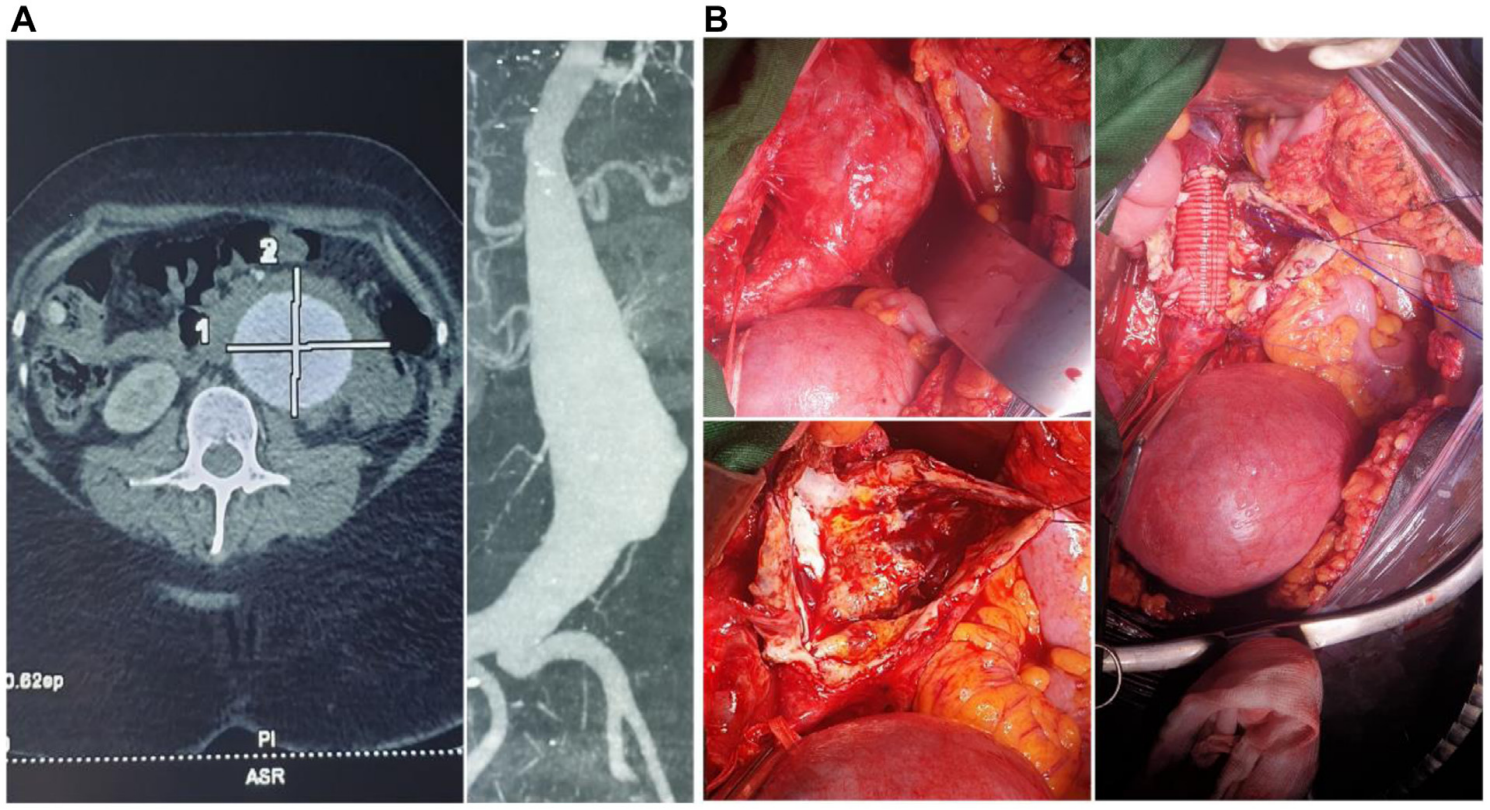
**(A)** Axial view of angio-computed tomography (CT) scan showing a large abdominal aortic aneurysm (AAA) (left) and two-dimensional MPR reconstruction (right). **(B)** Intraoperative view of the AAA (upper left corner) before “mise-à-plat” (lower left corner) and interposition of a 22-mm Dacron graft (right).

A 37-year-old pregnant woman, trigravida and secundiparous, at 17 weeks of gestation, was referred to our center for a large and pulsatile abdominal mass. She complained of progressively increasing abdominal and back pain. Doppler ultrasound examination showed an infrarenal AAA of 61 mm in diameter. Upon admission, emergent computed tomography angiography revealed a fissured infrarenal aneurysm of 85 mm in diameter with a dilated supra and juxtarenal abdominal aorta (Fig 1, *A*). She had no past medical history except an increased body mass index at 30. Of note, her phenotype was suggestive of Marfan syndrome (tall with long limbs, scoliosis, and aortic aneurysm). There was no sign of infection.

**Fig 2:**
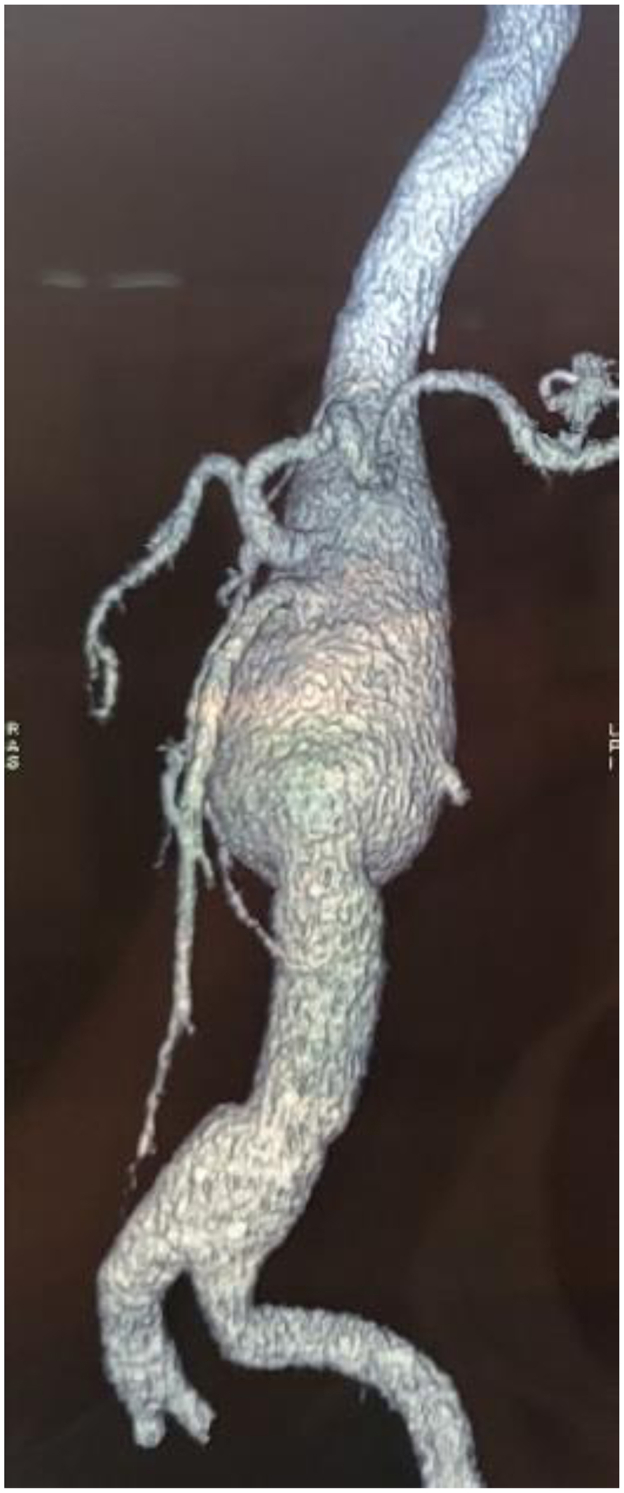
Six-month postoperative angio-computed tomography (CT) scan showing a patent graft and significant enlargement of the visceral aorta above the reconstruction.

The patient received tocolytic treatment and proceeded to open surgery at 18 weeks of gestation. The procedure consisted in open surgical repair with a 60-minute infrarenal cross-clamping and interposition of a 22-mm Dacron aortic graft (Fig 1, *B*). Cross-clamping was extended owing to hemodynamic instability that required appropriate vascular filling. Serial fetal echocardiography examinations were performed in the immediate postoperative course to monitor the fetus’ vitality. The postoperative period was uneventful, and she was discharged at day 10. She gave birth at term to a healthy male baby 4 months later. Nine months after her initial surgery, she was readmitted because of abdominal pain. Doppler ultrasound examination and angio-computed tomography both revealed a rapid increase of the juxtarenal and visceral part of the aorta at 52 mm in diameter (Fig 2). She was deemed fit for both open and endovascular (branched endovascular aneurysm repair) surgery, which she cannot afford yet. The patient gave her full consent for publication of this case.

## Discussion

Reports of treatment of AAA during pregnancy are scarce.^1^ Its prevalence in young individuals is <1%. Its association with pregnancy is associated with a stark prognosis.^1^^,^^6^^,^^7^ Hemodynamic, histological, and hormonal changes associated with pregnancy greatly impact the aortic wall and significantly increase the risk of rupture.^1^ Marfan syndrome and other collagen-associated disorders represent the main causes described in this context.^2^^,^^3^ The present patient presented a Marfan phenotype. Although aneurysms in patients with Marfan syndrome typically involve the ascending aorta,^4^^,^^8^^,^^9^ the abdominal aorta may also be affected. Unfortunately, owing to financial issues, genetic testing was not performed. On admission, she presented with intense pain and a large diameter aneurysm constituting a formal indication for treatment. Open surgery was favored over endovascular treatment because the anatomy of the aorta was unfavorable, because of the ongoing pregnancy and the concern regarding irradiation and because of the likely context of Marfan syndrome.^4^^,^^10^^,^^11^ Also, fenestrated endovascular aneurysm repair, in particular in an emergent situation, is not performed routinely in our institution owing to technical and economic concerns, although others may have favored this latter option.^10^ Tocolytic treatment was performed before open surgery. This case highlights that multidisciplinary treatment in referral centers is mandatory for these challenging cases and can result in good outcomes. In the absence of any septic event, the rapid enlargement of her proximal inter-renal and visceral aorta during follow-up supports the likelihood of collagen disease. Unfortunately, the required treatment indicated for this patient is not currently possible in our setting owing to technical and financial constraints. This patient and many others with more common arterial disease are pending vascular care. We hope this case can alert regarding the critical need to establish modern vascular surgery centers to cope with the epidemiological and demographical challenges in Western Africa.

## Disclosures

None.
